# Totally laparoscopic treatment of intestinal tuberculosis complicated with bowel perforation: The first case report in worldwide literature with a brief review

**DOI:** 10.3389/fsurg.2022.956124

**Published:** 2022-08-09

**Authors:** Giuseppe Di Buono, Roberta Vella, Giuseppe Amato, Giorgio Romano, Vito Rodolico, Marta Saverino, Giovanni De Lisi, Giorgio Romano, Salvatore Buscemi, Antonino Agrusa

**Affiliations:** ^1^ Department of Surgical, Oncological and Oral Sciences, University of Palermo, Palermo, Italy; ^2^ Department of Health Promotion Sciences Maternal and Infantile Care, Internal Medicine and Medical Specialities (PROMISE), University of Palermo, Palermo, Italy

**Keywords:** peritonitis, abdominal tuberculosis, bowel perforation, laparoscopy, intracorporeal anastomosis

## Abstract

**Introduction:**

Bowel perforation is a relatively rare presentation of abdominal tuberculosis, whose diagnosis is challenging but fundamental to minimize morbidity and mortality. Laparoscopy is considered an effective modality for diagnosis, but its role in surgical treatment is still not established. We reported the first worldwide case of totally laparoscopic treatment of intestinal tuberculosis complicated with bowel perforation.

**Case presentation:**

A 30-year-old man with a history of weight loss, preceded by 2 years of nonproductive cough, was admitted to the Infectious Disease Department with a presumed diagnosis of tuberculosis. A microbiological culture test confirmed the diagnosis, and the patient undertook quadruple antituberculous therapy. During hospitalization, he presented sudden abdominal pain, fever, and vomit. An abdominal CT scan showed small bowel perforation with granulomatous reaction. Laparoscopy was performed and revealed a 2 cm perforation on the medium ileum. Small bowel resection and totally intracorporeal side-to-side anastomosis were performed. No complication occurred until a clinical follow-up of 2 months.

**Conclusion:**

In consideration of the increasing incidence of intestinal TB in both underdeveloped and Western countries, the diagnosis of this pathology should be taken into account in high-risk patients. Probably, the diagnostic challenges and emergency settings of intestinal TB with perforation and peritonitis, together with the lack of standardized guidelines regarding surgical management, make the use of laparoscopy apparently arduous, but the known advantages of laparoscopy and its technical feasibility should make it a conceivable option for the treatment of complicated cases.

## Introduction

Tuberculosis (TB) is an infectious disease caused by *Mycobacterium tuberculosis*, a member of the M. tuberculosis complex. It is a major global health concern since more than 1.7 billion people (approximately 22% of the world population) are estimated to be infected with *M. tuberculosis* (1). According to the World Health Organization (WHO), the global incidence of TB peaked around 2003 and appeared to be declining slowly (2). COVID-19 pandemic (3) has led to a large decrease in the number of people newly diagnosed with TB, while the number of people who died from TB increased.

TB is a multisystem disease that primarily affects the lungs [referred to as pulmonary TB (PTB)]; however, it can affect any organ of the body wherein it is termed extrapulmonary TB (EPTB) (4). According to WHO, PTB and EPTB comprise 84% and 16% of all TB cases, respectively (5). The incidence of the forms of EPTB varies among countries, with the most commonly reported sites being the lymphatic, skeletal system, and pleura (6–10), followed by the urinary system, digestive system, cerebrospinal meninges, and breast (11). In Europe, abdominal tuberculosis is the sixth most prevalent presentation of extrapulmonary tuberculosis, comprising around 5% of all cases of TB worldwide (12). The relevance of this rare manifestation of TB has recently increased due to the influx of immigration and the prevalence of HIV infection, which are the main risk factors for the development of abdominal TB (4).

Abdominal involvement can occur during primary infection or following secondary reactivation. For instance, extrapulmonary tuberculosis, such as appendicular tuberculosis (ATB) and thyroid tuberculosis, has been described even in the primary form without any evidence of the disease elsewhere (13, 14).

Routes of infection of the gastrointestinal tract include ingestion of infected milk or sputum, hematogenous spread from distant tubercular focus, or contagious from infected adjacent foci and through lymphatic channels (12). Even though several internal organs can be involved, the most common sites include the peritoneum, bowel, especially the ileocecal area and/or lymph nodes (15). Among clinical manifestations that can occur in intestinal TB, including abdominal pain, diarrhea, lower gastrointestinal bleeding, constipation, and constitutional symptoms (fever, malaise, night sweats, anorexia, and weight loss), several complications figure an acute abdomen that could require surgical intervention, such as fistula, intestinal strictures (16), bowel obstruction, intussusception (17), and perforation (18, 19). Bowel perforation is an uncommon complication of intestinal TB that may occur more commonly in hypertrophic variety due to the reactive thickening of the peritoneum and subsequent adhesion formations that may subsequently perforate (20). Moreover, intestinal perforation has been described as a paradoxical reaction to antitubercular therapy (21). Although the pathophysiological mechanism is not fully understood, it is believed that a decrease in bacterial load would stimulate a host delayed-type hypersensitivity response with subsequent tissue damage (22). The diagnosis of intestinal TB could be challenging since its clinical manifestations and radiologic features can mimic other diseases, especially Crohn's disease (23). Relevant epidemiologic factors (endemic area, immunocompromised patients) and a high index of suspicion by the clinician need to be maintained to make the appropriate diagnosis. CT is the most helpful imaging modality to evaluate abdominal TB. It can show the site and extent of the inflammatory process. Chest radiography should be performed to support the diagnosis of TB, even though a normal chest x-ray does not exclude the disease (24). Definitive diagnosis can be established by demonstration of *M. tuberculosis* in peritoneal fluid, a biopsy specimen of an involved site, obtained through laparoscopy (sensitivity and specificity of 93% and 98%, respectively (25, 26), or endoscopy, or *via* mycobacterial culture and/or nucleic acid amplification test (NAAT) (27–29). On microscopic examination, three gross forms of intestinal tuberculosis are classically described: ulcerative, hypertrophic, and ulcerohypertrophic (12, 15, 20, 30). However, histologic features, like the presence of caseating granulomas, with or without demonstration of acid-fast bacilli, are suggestive of TB but are not pathognomonic (21, 22, 29). We report a case of intestinal tuberculosis complicated by ileal perforation with concurrent peritonitis, which was treated surgically through a laparoscopic approach. To our knowledge, this is the first case in worldwide literature of totally laparoscopic treatment of bowel perforation due to gastrointestinal tuberculosis. An appropriate diagnostic pathway was followed, including laboratory, instrumental and intraoperative findings. This case report has been reported in line with the CARE guidelines.

## Patient information

A 30-year-old man of Chinese origin was admitted to the Infectious Diseases Department of our University Hospital with a 2 month history of progressively weight loss, preceded by 2 years of nonproductive cough. No other diseases are known in the patient’s past history. Unfortunately, linguistic barriers made communication with the patient difficult, and no further information regarding his history, including family and psychosocial history, could be obtained.

## Clinical findings

On admission, the patient was cachectic (BMI 16.5) and had a high respiratory rate (RR 34/min), fever (body temperature 38.5 °C), and nonproductive cough with thoracic pain. Sars-Cov-2 nasopharyngeal swab test resulted negative. Microbiological tests for HIV and hepatitis viruses resulted negative.

## Timeline

**Figure 1:**
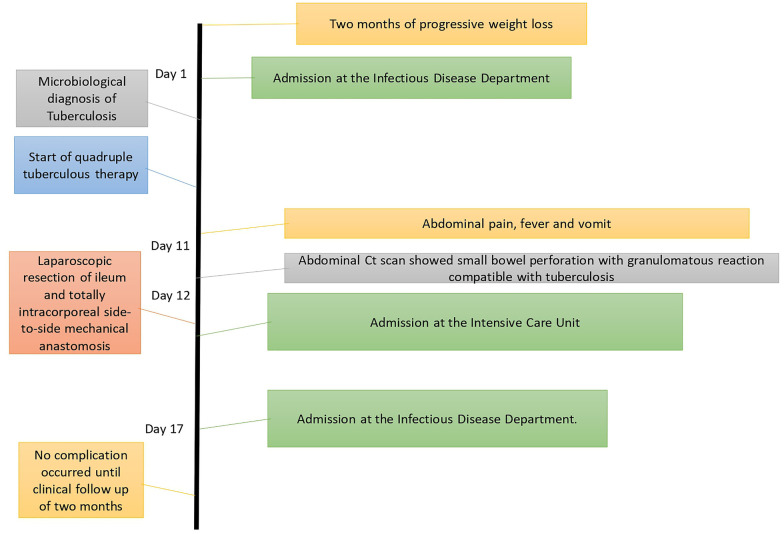
Timeline of diagnostic assessment, procedures, and outcomes.

In Figure 1, we describe the timeline of diagnostic assessment, surgical procedure, and outcome.

## Diagnostic assessment

**Figure 2:**
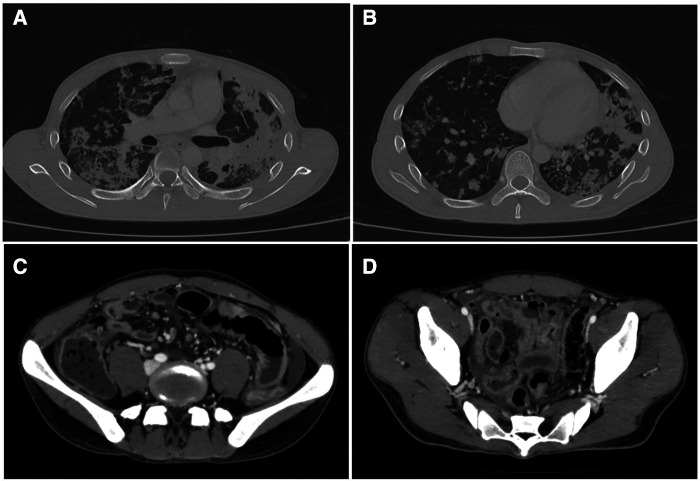
(**A,B**) Thoracic contrast-enhanced CT scan showing multiples areas of consolidation with cavitation (maximum diameter 35 mm) and mediastinal lymphadenopathy. (**C,D**) Abdominal CT scan showing small bowel perforation with granulomatous reaction compatible with tuberculosis.

The antituberculosis test, including Ziehl–Neelsen stain on sputum smear examination, TB-PCR, and the microbiological culture test resulted positive. The patient started a quadruple antituberculous therapy (rifampicin 600 mg; isoniazid 300 mg; pyrazinamide 1,500 mg; ethambutol 1,200 mg). Imaging evaluation through thoracic and abdominal contrast-enhanced CT scanning showed multiple areas of consolidation with cavitation (maximum diameter 35 mm) and mediastinal lymphadenopathy (Figures 2A,B). The blood culture test resulted negative. On the 11th day of hospitalization, the ­patient presented sudden lower abdominal pain, vomit, and hyperpyrexia. On examination, he appeared debilitated, had a high respiratory rate (RR 40/min) and a high cardiac rate (HR 115 bpm), and the abdomen was rigid, with generalized tenderness. Bowel sounds were absent. In the laboratory tests, hemoglobin was 11.9 g/dl and the white blood cell (WBC) count was 29.39×10^3^/µl. An abdominal CT scan showed small bowel perforation with granulomatous reaction compatible with intestinal tuberculosis (Figures 2C,D)

## Therapeutic intervention

**Figure 3:**
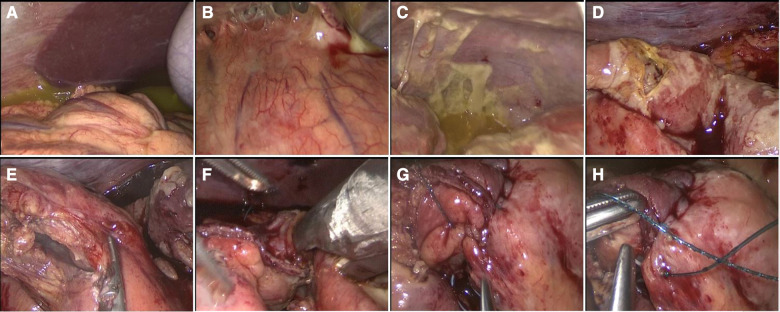
(**A–C**) Laparoscopic exploration with generalized purulent peritonitis with enteric fluid and granulomatous reaction. (**D**) Medium ileal 2 cm perforation. (**E**) Laparoscopic resection with the use of the Harmonic ACE device. (**F**) Intracorporeal side-to-side anastomosis. (**G,H**) Closure of enterotomy with double line continuous barbed suture (V-Loc 3-0, Medtronic).

With a preoperative clinical and radiological diagnosis of small bowel perforation secondary to likely intestinal tuberculosis, written informed consent for emergency surgery was obtained. Surgery was performed by a young surgeon. Regarding the surgical approach, despite marked distension of the stomach and small bowel with multiple air-fluid levels, laparoscopy was considered a conceivable option. The patient was positioned supine with both arms tucked. The surgeon and camera operator were located on the patient's left side. A urinary catheter was placed. Pneumoperitoneum was induced through an open trans-umbilical technique with the placement of a 12 mm optical trocar (31). Other two 5 mm ports were inserted, one in the suprapubic region and one in the left flank like in laparoscopic appendectomy (32). Laparoscopic exploration revealed conspicuous purulent peritoneal fluid that was sent for microbiology examination and dilation of small bowel loops that reduced the size of the operating space (Figure 3). The laparoscopic maneuvers were very difficult because of the inflammation and multiple severe adhesions of the medium ileum. A 2 cm perforation on the medium-terminal ileum was identified. We performed a resection of a tract of 20 cm of medium ileum, including the site of perforation, using ultrasonic devices (Harmonic ACE+7 Shears with Advanced Hemostasis) and a linear surgical stapler (Echelon Flex 45 mm, white reload), followed by totally intracorporeal side-to-side mechanical anastomosis (Echelon Flex 45 mm, white reload). Stapler-access enterotomy was closed through double line continuous barbed suture (V-Loc 3–0, Medtronic) (33). Exploration of intestinal loops, from ileocecal valve to Treitz ligament, did not evidence further macroscopically detectable lesions. Irrigation and suction with warm saline solution (around 5 L) were conducted, and a drain was placed in the pelvic cavity.

## Follow-up and outcome

**Figure 4:**
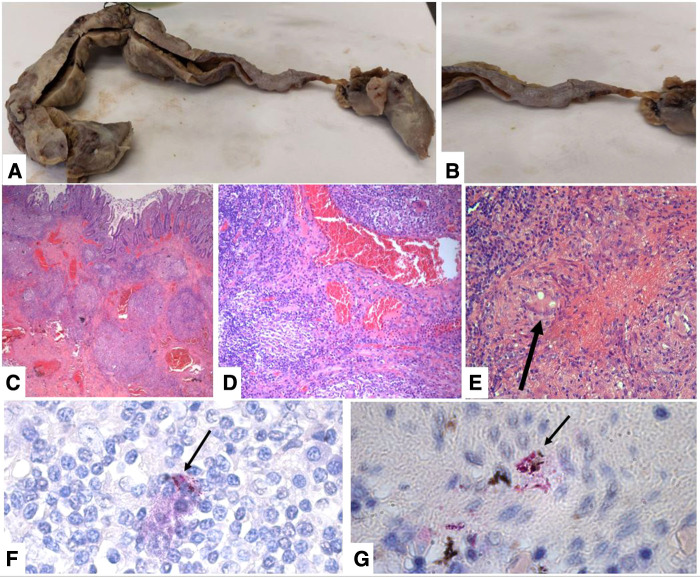
(**A**) macroscopic view of the ileum after formalic fixation. (**B**) Particular of the stenotic tract covered by fibrinous exudate. (**C**) Transmural inflammatory infiltrate with multiple caseating granulomas and vascular stasis (H&E stain, original magnification 40×). (**D**) Granulomatous chronic inflammation destroy glandular elements (H&E stain, original magnification 100×). (**E**) Caseous granuloma with a Langhans multinucleated cell (H&E stain, original magnification 200×). (**F–G**) Ziehl–Neelsen staining showing multiple diffuse acid-fast bacilli fluctuating in the stroma and sometimes phagocytosed by histiocytes (arrows) (Ziehl–Neelsen stain, original magnification 1000×).

After surgery, the patient was admitted to the intensive care unit (ICU), and on the first postoperative day (POD), the patient was successfully extubated and oral feeding was started. On the fifth POD, we transferred the patient to the Infectious Disease Department. Peritoneal fluid culture found *Enterococcus faecium* and *Streptococcus gordonii*; therefore, intravenous antibiotic therapy with meropenem (1 g every 8 h) and quadruple antituberculous treatment (rifampicin 600 mg single daily administration; isoniazid 300 mg single daily administration; pyrazinamide 1,500 mg single daily administration; ethambutol 1,200 mg every 12 h) were administered. No complications occurred until a clinical follow-up of 2 months. The pathological macroscopic examination showed that the ileum wall was widely thickened with a stenotic tract covered by fibrinous exudate and brownish-gray color; two areas of perforation were found, one of 2 × 1.2 cm and one of 3 × 1 cm (Figures 3A,B). The histologic examination showed multiple mucosal erosions with a transmural acute and chronic inflammatory cell infiltrate associated with the granulomatous reaction, multiple Langhans giant cells, and diffuse areas of caseous necrosis (Figures 4C–E). Vascular stasis and blood congestion have been found in the submucosa and muscularis tunicae. Ziehl–Neelsen staining confirmed the presence of acid-fast bacilli (Figures. 4F,G).

## Discussion

Although rare, there is an increasing incidence of abdominal tuberculosis in developed countries. The diagnosis of this disease continues to pose significant challenges, but early recognition is fundamental to minimize morbidity and mortality (34). The treatment is primary pharmacological, but surgical intervention is needed when complications occur or when the diagnosis is uncertain. It is reported that 20%–40% of abdominal tuberculosis cases present acutely and need emergency surgery (35–37). Surgeries performed include radical resections, enteroenterostomy or ileotransverse anastomosis (12), and conservative surgeries, such as strictureplasty and adhesiolysis (38). Being a systemic disease, surgery should be more conservative as possible and should consider the malnourishment of patients, especially when performing radical resections, and the risk of recurrence (37). Laparoscopy is considered an effective modality for diagnosis (39) since it allows differential diagnosis of diseases that can mimic gastrointestinal TB (Crohn's disease, lymphoma, and malignancies) and represents one of the main tools for obtaining biopsies (40). However, its role in the surgical treatment of complicated abdominal TB is still not established, and most of the cases of patients with gastrointestinal TB presenting with acute abdomen requiring emergency surgery report the use of emergency laparotomy. The use of laparoscopy in the treatment pathway of patients with suspected abdominal TB is described in a few articles, even though neither small bowel perforation nor totally laparoscopic anastomosis was reported. Mousa et al. (35), in their retrospective study, signaled that five out of thirteen patients with abdominal TB requiring surgical intervention were treated with laparoscopy and three were converted to open surgery. Entirely performed laparoscopic surgeries comprised only cholecystectomy and diagnostic biopsy. Souhaib et al. (41) described the use of laparoscopy for a 33-year-old female patient with suspected acute cholecystitis. Explorative laparoscopy revealed a duodenal ulcer, blocked by the gallbladder, caused by tuberculosis involvement and, after conversion to open surgery, cholecystectomy was performed. Sasse et al. (42) carried out laparoscopic surgery to perform a peritoneal biopsy and a protective ileostomy. To our knowledge, this is the first case report of intestinal perforation due to abdominal TB in which totally laparoscopic bowel resection and intracorporeal anastomosis were achieved, reporting satisfactory recovery on clinical follow-up. Coccolini et al. (43) conducted a retrospective review of cases of intestinal perforation due to abdominal TB published until 2009 and considered 54 articles, including 622 patients. The review highlighted the use of the laparoscopic approach only in an elective diagnostic setting, while its use in the emergency was not advised and its role in treatment was not proposed. Limitations encountered included the small size of the operating field, especially when multiple adhesions existed, thus preventing adequate laparoscopic inspection (44). Lack of evidence regarding the safeness and feasibility of the laparoscopic approach in the emergency setting, together with the lack of standardized guidelines regarding the surgical management of intestinal TB, could explain the reticence of surgeons, even if this approach could minimize hospital stay when it can be safely applied.

## Data Availability

The original contributions presented in the study are included in the article/Supplementary Material, further inquiries can be directed to the corresponding author.
